# Suggestions, in Relation to the Examination of Recruits for the Naval Service

**Published:** 1838-07-18

**Authors:** Thomas Williamson

**Affiliations:** Surgeon of the U. S. Navy


*Suggestions, in relation to the Examination of Recruits for the Naval Service.* By THOMAS WILLIAMSON, M. D., Surgeon of the U. S. Navy.

U. S. NAVAL HOSPITAL.
*Near Norfolk, Virginia*, June 26th, 1838.

*Gentlemen:*—As we are frequently called upon to examine persons for the naval service of the United States, I would suggest to such medical officers as have this duty to perform, the following specific instructions, viz.:

To guard the naval service more effectually from the introduction of improper persons into it, and to prevent the diseased, disabled, and worthless from being admitted, it is necessary that the following regulations be rigidly enforced by all medical officers, and others, at our various recruiting stations, and elsewhere, when recruits are required for the service.

1st. The applicant must be sober, perfectly naked, and, then, with the greatest care and attention, are to be examined the peculiar structure of his head, chest, abdomen, pelvis, and extremities, his respiration, pulse, vision, hearing, speech, smell, taste, and touch.

2d. All diseases, defects, and blemishes, that would disqualify a recruit from being placed instantly on laborious duty, must prevent his being received into the service; such as the loss of a limb or limbs, distortions of the bones, caries, exostosis, nodes, necrosis, dislocations, extensive fractures, great deficiency of teeth, old age, loss of toes, or fingers, rigidness, or disease of the joints, loss of motion, or sensation in any of the muscles, derangement of the brain, or nervous system, extensive abscesses, ulcers, tumors, fistulæ, chronic diseases of the mucous membranes, venereal affections, diseases of the chest, scrofula, and all other diseases of the glandular system, visceral obstructions, herniæ, dropsies, enlarged abdomen, organic affections of the heart and arteries, varicose veins, marks of old ulcers, unhealthy appearance of the shins, and cutaneous eruptions.

3d. Defects:—the loss of an eye, ear, or part thereof, the organs of generation, finger or fingers, toe or toes, and other deficiencies that are palpably so, must be rejected. Blemishes that are unsightly, such as marks of punishment, extensive scalds, and burns, wounds, hare-lip, extensive cicatrices, are also to be rejected.

If the above directions are attended to, the disadvantages resulting to the naval service, would soon cease, and we should not hear our commanders and surgeons complaining so often of the unfitness of the men shipped for the naval service. A great difficulty has arisen with us all in not having some specific instructions to direct us in the examination of recruits, much being left to our discretion. The consequence has been, that some surgeons will pass a man with a disease, whilst others would reject him. The question arises, whether any one shall be received into the naval service who has any disease upon him? I should unhesitatingly answer that he should not. If men are to be rejected on account of disease, what are the diseases which ought to cause them to be rejected? Are defects and blemishes to be rejected? I answer, yes, as much so as if they were diseased. I therefore, gentlemen, believe, that if the directions which are hinted at in this, are followed, a happy result will be the consequence.

THOMAS WILLIAMSON.

To the Editors of the Medical Examiner.

